# Human Cumulus Cells in Long-Term In Vitro Culture Reflect Differential Expression Profile of Genes Responsible for Planned Cell Death and Aging—A Study of New Molecular Markers

**DOI:** 10.3390/cells9051265

**Published:** 2020-05-21

**Authors:** Błażej Chermuła, Wiesława Kranc, Karol Jopek, Joanna Budna-Tukan, Greg Hutchings, Claudia Dompe, Lisa Moncrieff, Krzysztof Janowicz, Małgorzata Józkowiak, Michal Jeseta, Jim Petitte, Paul Mozdziak, Leszek Pawelczyk, Robert Z. Spaczyński, Bartosz Kempisty

**Affiliations:** 1 Division of Infertility and Reproductive Endocrinology, Department of Gynecology, Obstetrics and Gynecological Oncology, Poznan University of Medical Sciences, 33 Polna St., 60-535 Poznan, Poland; blazej.chermula@wp.pl (B.C.); pawelczyk.leszek@ump.edu.pl (L.P.); rspaczynski@yahoo.com (R.Z.S.); 2 Department of Anatomy, Poznan University of Medical Sciences, 6 Swiecickiego St., 60-781 Poznan, Poland; wkranc@ump.edu.pl (W.K.); g.hutchings.16@abnd.ac.uk (G.H.); krzysztof.janowicz.16@abdn.ac.uk (K.J.); 3 Department of Histology and Embryology, Poznan University of Medical Sciences, 6 Swiecickiego St., 60-781 Poznan, Poland; karoljopek01@gmail.com (K.J.); joanna.budna@wp.pl (J.B.-T.); claudia.dompe.16@abdn.ac.uk (C.D.); l.moncrieff.16@abdn.ac.uk (L.M.); 4 The School of Medicine, Medical Sciences and Nutrition, University of Aberdeen, Aberdeen AB25 2ZD, UK; 5 Department of Toxicology, Poznan University of Medical Sciences, 30 Dojazd St., 60-631 Poznan, Poland; malgorzata.jozkowiak@gmail.com; 6 Department of Obstetrics and Gynecology, University Hospital and Masaryk University, 20 Jihlavská St., 62500 Brno, Czech Republic; jeseta@gmail.com; 7 Prestage Department of Poultry Science, North Carolina State University, Raleigh, NC 27695, USA; jnppo@ncsu.edu; 8 Physiology Graduate Program, North Carolina State University, Raleigh, NC 27695, USA; pemozdzi@ncsu.edu; 9 Department of Veterinary Surgery, Institute of Veterinary Medicine, Nicolaus Copernicus University in Torun, 1 Lwowska St., 87-100 Torun, Poland

**Keywords:** cumulus cells, human, programmed cell death, gene expression

## Abstract

In the ovarian follicle, maturation of the oocyte increases in the presence of somatic cells called cumulus cells (CCs). These cells form a direct barrier between the oocyte and external environment. Thanks to bidirectional communication, they have a direct impact on the oocyte, its quality and development potential. Understanding the genetic profile of CCs appears to be important in elucidating the physiology of oocytes. Long-term in vitro culture of CCs collected from patients undergoing controlled ovarian stimulation during in vitro fertilization procedure was conducted. Using microarray expression analysis, transcript levels were assessed on day 1, 7, 15, and 30 of culture. Apoptosis and aging of CCs strictly influence oocyte quality and subsequently the outcome of assisted reproductive technologies (ART). Thus, particular attention was paid to the analysis of genes involved in programmed cell death, aging, and apoptosis. Due to the detailed level of expression analysis of each of the 133 analyzed genes, three groups were selected: first with significantly decreased expression during the culture; second with the statistically lowest increase in expression; and third with the highest significant increase in expression. *COL3A1*, *SFRP4*, *CTGF*, *HTR2B*, *VCAM1*, *TNFRSF11B* genes, belonging to the third group, were identified as potential carriers of information on oocyte quality.

## 1. Introduction

Appropriate oocyte selection for intracytoplasmic sperm injection (ICSI), as a part of in vitro fertilization (IVF) procedure, is mainly based on their morphological assessment. In recent times, evaluation of predictive factors such as follicular fluid markers, as well as genetic and epigenetic properties of cumulus cells (CCs) have become promising in appropriate oocyte selection [1,2].

In the growing ovarian follicle, communication between oocyte and surrounding somatic cells (CCs) is a complicated process. Both CCs and the oocyte actively regulate the bidirectional communication [3,4]. By exchanging molecular signals, CCs communicate permanently with the oocyte, acquiring new properties appropriate for the follicular growth phase. Through their cytoplasmic protrusions, CCs penetrate oocyte zona pellucida, connect to its cell membrane, and form specialized channels called gap junctions (GJs). One of the basic CC functions is the restraining of meiosis and cytoplasmic oocyte maturity, achieved through transmitting molecular signals inwards to the oocyte in the form of metabolites such as AMP or Ca^2+^ ions. Growth, differentiation and proliferation factors are secreted and transported from the oocyte to CCs. This exchange is carried out precisely through the GJs, connecting their cell membranes with each other [5,6]. Larger molecules such as RNA are also participants of this CC to oocyte transport [7,8]. This fact has been already described many times, but specific functions and transport mechanism are still not fully understood [5]. Furthermore, GJs play an important role in programmed cell death (PCD) regulation [9]. It is presumed that PCD and CC aging strictly influence the oocyte quality and thus, may be regarded as a new predictors of assisted reproductive technologies (ART) efficacy [2,10].

Programmed cell death is a process that eliminates unnecessary and damaged cells. PCD in multicellular organisms allows physiological maintenance of homeostasis. It is a complex, genetically regulated process during which the cell launches molecular and physiological mechanisms leading to its degradation. PCD is induced by extracellular signals that control the proper cell functioning. It is a process that accompanies whole ontogenesis [11,12]. To date, more than 150 unique genes involved in the prevention of PCD have been described in the available literature [13]. PCD plays the most important role during gametogenesis, various stages of embryogenesis, organogenesis, and tissue renewal, as well as during the elimination of abnormal cells [14,15]. Regulation of oocyte maturation and follicular growth includes a cascade of subsequent, specific events. CC communication disorders, leading to diminished oocyte quality, may be caused by disturbances in cumulus cells’ aging and apoptosis processes [14].

Microarray expression technique makes it possible to study and identify new cellular molecular markers and to analyze functions of newly identified transcripts in the examined cells or tissue [16,17,18,19,20,21]. The use of microarrays in the analysis of granulosa cell (GC) gene expression enabled identification of gene ontology (GO) groups involved in oocyte development and apoptosis [22,23,24].

The main goal of this study was to analyze, in human CCs, expression of genes belonging to: “Cell death”, “Programmed cell death”, “Regulation of cell death”, “Positive regulation of apoptotic process”, “Positive regulation of cell death”, “Extrinsic apoptotic signaling pathway”, “Negative regulation of cell death”, “Negative regulation of apoptotic process”, and “Aging” GO groups during long-term in vitro culture (IVC). Our main goal was to identify and describe new molecular markers involved in the programmed death, especially apoptosis and aging processes, both in cumulus cells and indirectly in oocytes.

Based on previous work [25,26], it appears that evaluation of CC gene expression involved in programmed cell death, especially apoptosis and aging may be a predictor of oocyte quality and potentially included in the routine IVF procedures. Obtaining the complete CC transcriptomic profile may help to foresee oocyte development potential for successful fertilization and embryo formation. The current research can be helpful in developing accurate and non-invasive tools for predicting ART effectiveness. Presented studies are the first step to identify CC genes that can be used to determine oocyte potential for further fertilization with the use of ART techniques. One of the most important aspects of non-invasive oocyte assessment is the determination of molecular markers for selection of the most appropriate oocytes for further procedures. Additionally, CC long-term in vitro cultures can also show us the directions in which they can differentiate in laboratory conditions. It also seems interesting to correlate growth and differentiation of these cells in in vitro conditions with their in vivo behavior during corpus luteum formation.

## 2. Materials and Methods

### 2.1. Patients Characteristics

Human CCs were obtained from 12 infertile female patients (mean age 33.67 years ± 1.46 (SEM; standard error of the mean), range 25–40) undergoing IVF-ICSI procedure at the Centre of Diagnosis and Treatment of Infertility at Division of Infertility and Reproductive Endocrinology, Poznan University of Medical Sciences. Controlled ovarian hyperstimulation was induced by the administration of recombinant human follicle-stimulating hormone (rhFSH; Gonal F, Merck sp. z o.o, Poland or Puregon, MSD Poland sp. z o.o, Poland) and highly purified human menopausal gonadotropin (hMG; Menopur, Ferring Pharmaceuticals Poland sp. z o.o, Poland) in individualized doses in gonadotropin releasing hormone (GnRH) antagonist protocol (Cetrotide, cetrorelix 0.25 mg, Merck sp. z o.o, Poland or Orgalutran, ganirelix 0,25 mg, MSD Poland sp. z o.o, Poland). Ovulation was induced with rh chorionic gonadotropin (rhCG; Ovitrelle, 250 ug, Merck sp. z o.o, Poland) to trigger oocyte maturation on days 9 to 12 after initial gonadotropin administration. Oocyte retrieval was performed by transvaginal ultrasound 36 h after rhCG administration. Patients with known tubal infertility factor were selected for this study. The selected female infertile patients had no history of ovarian surgery, no other chronic or endocrine diseases, and a BMI < 30 kg/m^2^. We excluded patients with polycystic ovarian syndrome (PCOS), endometriosis, premature ovarian insufficiency (POI), and with poor ovarian response.

This research has been approved by Poznan University of Medical Sciences Bioethical Committee with 1290/18 resolution.

### 2.2. IVF Patients’ Cumulus Cells Collection

After oocyte pick-up (OPU), embryologists selected oocyte-cumulus complexes (COCs) for further IVF procedure. According to a routine procedure of preparing an oocyte for fertilization by ICSI, denudation was performed. The process consisted of mechanical and enzymatic (800 IU/mL of HYASE-10X) removal of the surrounding oocyte corona radiata and cumulus oophorus somatic cells forming the COC. On average, 10 COCs were obtained from each patient. After complete denudation, oocyte CCs were pooled from individual patients and cultured together. A separate 30-day in vitro culture was carried out for CCs obtained from each patient.

### 2.3. Long-Term Primary In Vitro Culture

Collected and grouped CCs were washed twice with culture medium and centrifuged at 200× *g* for 10 min at RT. Culture medium consisted of DMEM (Dulbecco’s Modified Eagle’s Medium, Sigma; Merck KGaA, Darmstadt, Germany) supplemented with 10 mg/mL gentamicin (Invitrogen; Thermo Fisher Scientific, Inc.), 2% fetal bovine serum FBS (FBS; Sigma; Merck KGaA), 4 mM l-glutamine (stock 200 mM, Invitrogen; Thermo Fisher Scientific, Inc., Waltham, MA, USA), 10,000 U/mL penicillin and 10,000 μg/mL streptomycin (Invitrogen; Thermo Fisher Scientific, Inc.). The cells were then counted using the “Neubauer improved” counting chamber (ISO LAB Laborgerate GmbH, DIN Certificate EN ISO 9001). Only the samples in which cells showed a viability of over 90% were used for further culture.

The study based on long-term (30 days) primary in vitro cultures. Four time intervals were investigated: moment 0 (24 h), corresponds approximately to the physiological properties of cells, while the following days show the changes that occur in the cultures; the 7th day of in vitro culture defines short-term culture; the 15th day of culture shows effects of the first passage; the 30th day of culture is the end of long-term in vitro culture.

CCs were cultured at 37 °C in humid 5% CO_2_ atmosphere. After reaching 90% confluence, cells in the culture were detached from the bottom of the 6-well culture plate culture by 1–2 min incubation with 0.05% trypsin-EDTA (Invitrogen; Thermo Fisher Scientific, Inc). CC culture lasted 30 days. The medium was changed every three days of culture. The cells were harvested on day 1, 7, 15, and 30 of culture. Samples derived from each patient were cultured separately, RNA material was pooled before microarray and RT-qPCR analysis.

### 2.4. Total RNA Isolation

After harvesting cells on day 1, 7, 15, and 30 of culture, total RNA was isolated. The process of isolation was performed according to modified Chomczyński and Sacchi method [27]. Briefly, CCs were suspended in 1 mL of monophasic guanidine thiocyanate and phenol solution (TRI Reagent^®^, Sigma; St. Luis, MO, USA; Merck KGaA). In the next step, chloroform was added to separate the phases, with the whole samples centrifuged afterwards. The upper, aqueous phase containing isolated RNA was collected. RNA was extracted using 2-propanol (Sigma; Merck KGaA, catalog number I9516), added in an amount adequate for 1 mL of TRI-reagent. Finally, the RNA was washed with 75% ethanol, dried, resuspended in 100µl of pure water and measured for concentration and purity. The total amount of mRNA was determined based on optical density at 260 nm, RNA purity was estimated using 260/280 nm absorption ratio (NanoDrop spectrophotometer, Thermo Scientific, Warsaw, Poland). Samples with a 260/280 absorbance coefficient greater than 1.8 were used for further experiments.

### 2.5. Microarray Expression Analysis

Previously prepared total RNA (100 ng) from each pooled sample were subjected to two rounds of sense cDNA amplification (Ambion^®^ WT Expression Kit, Ambion, Austin, TX, USA). The obtained cDNA was used for biotin labeling and fragmentation by Affymetrix GeneChip^®^ WT Terminal Labeling and Hybridization (Affymetrix, Santa Clara, CA, USA). Biotin-labeled fragments of cDNA (5.5 μg) were hybridized to the HG-U219 Strip (48 °C/20 h). The next step, the microarrays were washed and stained according to the provided protocol, using the Affymetrix GeneAtlas Fluidics Station. The microarrays were scanned using the GeneAtlas imaging station. Using the Affymetrix GeneAtlas^TM^ Operating Software (v.2.0.0.460, Affymetrix, Santa Clara, CA, USA), a preliminary analysis of the scanned systems was performed. The quality of gene expression data was confirmed in accordance with the quality control criteria provided by the software. The obtained CEL files were transferred to the data analysis software.

The analyses and obtained charts were created using the Bioconductor software and R programming language. The CEL file was merged with the description file. Using the Robust Multiarray Averaging (RMA) algorithm, the background was improved, normalized, and the results summarized. To determine the statistical significance of the analyzed genes, moderate t statistics from Bayes’ empirical method were performed. The *p*-value was corrected for multiple comparisons using the Benjamini and Hochberg false discovery indicator. The selection of significantly changed genes was based on *p*-values less than 0.05 and expression over two-fold.

The list of differentially expressed genes has been imported into the DAVID software, with complete genome selected as the default background [28]. Then, gene ontology (GO) domains were separated. The expression of the analyzed genes was subjected to a hierarchical clustering procedure, necessary for heatmap presentation.

In the next stage, we analyzed the relationship between genes belonging to selected GO terms using the GOplot package [29]. The obtained *z*-score resulted from up-regulated genes minus down-regulated genes divided by the square root of the count. Thanks to these calculations, it was possible to determine the course of changes of each gene ontology term.

Moreover, to predict the interactions between individual genes, received sets of genes from the selected GO BP terms were loaded into the STRING software (Search Tool for the Retrieval of Interacting Genes). Thanks to the STRING database, we were able to obtain information on protein/gene interactions. This data was developed on the basis of experimental methods, computational forecasting, and publication collections.

Using the Reactome FIViz application in Cytoscape 3.6.0 software, interactions between genes belonging to the selected GO BPs were analyzed. Reactome FIViz allows to find pathways and patterns of networks related to cancer and other diseases. This application has access to the Reactome Functional Interaction (FI) network, a highly reliable, manually selected pathway based on functional interaction protein network covering over 60% of human proteins.

### 2.6. Reverse Transcription Quantitative PCR Analysis (RT-qPCR)

RT-qPCR was performed to confirm the results obtained with microarray analysis. From each analyzed heatmap, three genes were selected: those with the highest, lowest, and intermediate levels of expression. Subsequently, using three biological samples in triplicate, the changes in their expression were analyzed. Reverse transcription was performed according to the reagent protocol provided by the manufacturer—SABiosciences (Frederick, MD, USA; RT2 First Stand kit-330401), using a Verlerimer 96-well thermocycler. For each reaction, 1 µg of RNA transcript was used. Thermocycling conditions were as follows: Preincubation at 37 °C for 30 s; 3-step amplification (95 °C for 15 s, 58 °C for 15 s, 72 °C for 15 s) for 45 cycles; melting (95 °C for 60 s, 40 °C for 60 s, 70 °C for 1 s, 95 °C for 1 s); cooling at 37 °C for 30 s. Gene expression was analyzed using the 2^−ΔΔCq^ method.

For real-time PCR, the 7900HT FAST PCR Real Time System (Applied Biosystems; Thermo Fisher Scientific, Inc.), Master Mix RT2 SYBR^®^ Green ROX ™ qPCR (Qiagen Sciences, Gaithersburg, MD, USA), and sequence-specific primers were used (Table 1). Gene expression was calculated with relative quantification (RQ) method, using 3-phosphate glyceraldehyde dehydrogenase (*GADPH*), β-actin (*ACTB*), and hypoxanthine 1 phosphoribosyltransferase (*HPRT1*) genes as the reference. The RT-qPCR primers were designed using Primer3Plus software (version 0.4.0; Whitehead Institute for Biomedical Research, Massachusetts Institute of Technology, Cambridge, MA, USA).

### 2.7. Statistical Analysis

To calculate the results and perform statistical analysis, the Bioconductor program (www.bioconductor.org) and R programming languages (version R 3.5.1; www.r-project.org) were used, with the analysis itself based on moderated t statistics from the Bayes’ empirical method. The *p* value was corrected for multiple comparisons using the Benjamin and Hochberg false detection index. *p* < 0.05 was considered as a statistically significant difference. To establish statistical significance of the enriched GO terms and KEGG paths, the DAVID database software (v.6.8; david.ncifcrf.gov) and Benjamini methods were used. Each GO term and KEGG pathway was considered significantly enriched if they contained at least 5 differentially expressed genes with *p* < 0.05. RT-q PCR statistical analysis was performed with the use of the Real Statistics Resource Pack for MS Excel 2016 (Microsoft Corporation, Redmond, WA, USA).

## 3. Results

### 3.1. Cumulus Cells Morphology

**Figure 1:**
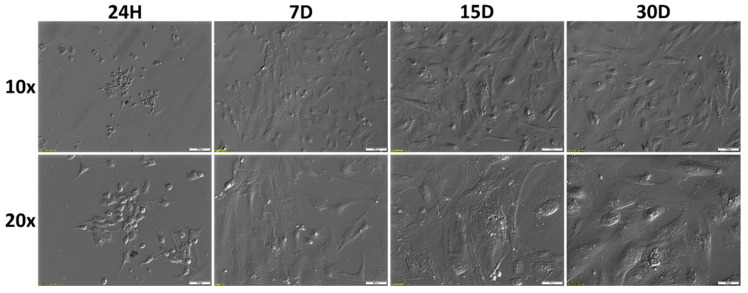
Changes in cumulus cells (GCs) morphology during long-term in vitro primary culture at individual time intervals. 24H: first day of culture; D: day of culture; 10×, 20×: magnification.

During 30 days of in vitro culture, morphological and potential phenotypic changes in cumulus cells were examined using a light microscope. Figure 1 presents the morphology of CC at individual time intervals of the primary in vitro culture. Changes in the morphology of CCs were observed at individual time intervals. CCs changed their shape from star-like to slightly elongated, spindle-like during primary in vitro culture. It was observed that CCs adhered very quickly to the flask bottom, just a few hours after transfer to the culture plate.

### 3.2. Profile of Gene Expression

Whole transcriptome profiling by Affymetrix microarray allowed us to analyze gene expression changes between 1 and the 7, 15, and 30 days of human CCs in vitro primary culture. Using human HgU 219 microarray, we examined expression of 49385 transcripts. Genes regarded as differentially expressed presented fold change higher than abs (2) and corrected *p*-value lower than 0.05 and consisted of 2169 different transcripts. All analyzed microarray data is available in the GEO database [30].

To retrieve gene ontology biological process terms (GO BP) containing differently expressed transcripts, the DAVID (Database for Annotation, Visualization, and Integrated Discovery) software was used. All up- and down-regulated genes were subjected to the DAVID search tool individually, with genes complying with previously defined criteria (adj. *p*-value < 0.05) selected. The analysis resulted in revealing differently expressed genes belonging to 657 GO BPs. The present paper focuses on 133 genes included in “cell death”, “programmed cell death”, “regulation of cell death”, “positive regulation of apoptotic process”, “positive regulation of cell death”, “extrinsic apoptotic signaling pathway”, “negative regulation of cell death”, “negative regulation of apoptotic process”, and “aging” GO BP terms. In the Supplementary Table S1, the expression of all 133 genes are presented. Table 2 summarizes gene symbols (only 16 genes with the largest change in expression), Entrez gene IDs, fold changes in expression ratio, corrected *p*-values, and mean value of fold change ratio of studied genes.

Among genes with in-culture decrease in expression, in the case of the *SFRP5*, *HMGCR*, *HMGB1,* and *ZC3H8* genes, uniform decrease was measured on 7, 15, and 30 days, when compared, which was made in 24 h culture as noted. In the case of *HMGCR* and *ARF6*, their even expression decrease was noted on days 7 and 15. On the 30th day, their decline in expression was at a lower level. In the case of genes in which an increase in expression was observed, *NLRP*, *TLE1*, *COL3A1*, *SFRP4,* and *TNFRSF1B* showed steady increase in subsequent measurements on day 7, 15, and 30. In the case of the *COL3A1,* compared to the measurement from day 7, in the last two weeks of the CCs culture there was a five-fold increase in expression of this gene. *ITSN1* expression was highest on 7th day of culture. In turn, *CTGF* and *VCAM* genes showed the highest increase in expression during second week of cultivation. After first week, *STK17A* and *HTR2B* genes showed an increase in expression in comparison to the measurement from the first day. Then, their expression on day 15 was lower, increasing again on day 30. It should be added that for the *HTR2B* gene the increase in expression measured on day 30 was ten times higher than that recorded on day 15. Discussed gene expression level changes on individual days of culture are listed in Table 1.

**Figure 2:**
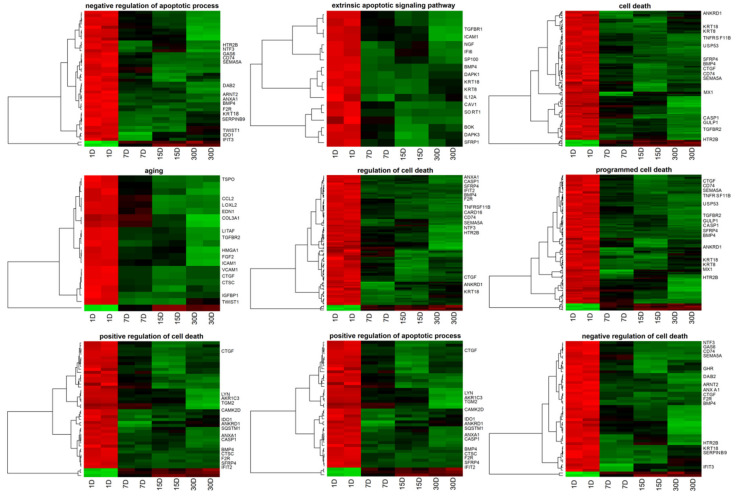
Heatmap representation of differentially expressed genes belonging to the “cell death”, “programmed cell death”, “regulation of cell death”, “positive regulation of apoptotic process”, “positive regulation of cell death”, “extrinsic apoptotic signaling pathway”, “negative regulation of cell death”, “negative regulation of apoptotic process”, and “aging” gene ontology biological process (GO BP) terms. Arbitrary signal intensity acquired from microarray analysis is represented by colors (green, higher; red, lower expression). Log2 signal intensity values for any single gene were resized to Row *z*-Score scale (from −2, the lowest expression to +2, the highest expression for single gene). The 15 genes most down-regulated and up-regulated genes are marked on each heatmap.

The 15 genes with the highest expression change were marked on each heatmap in Figure 2.

**Figure 3:**
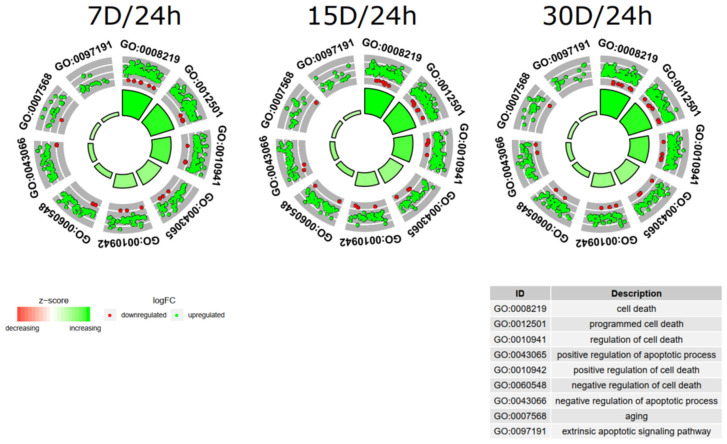
The circle plot showing the differently expressed genes and *z*-score of the “cell death”, “programmed cell death”, “regulation of cell death”, “positive regulation of apoptotic process”, “positive regulation of cell death”, “extrinsic apoptotic signaling pathway”, “negative regulation of cell death”, “negative regulation of apoptotic process”, and “aging” GO BP terms. The outer circle shows a scatter plot for each term of the fold change of the assigned genes. Green circles display up-regulation and red ones down-regulation. The inner circle shows the *z*-score of each GO BP term. The width of each bar corresponds to the number of genes within GO BP term and the color corresponds to the *z*-score.

Next, the enrichment of each GO BP term was calculated as *z*-score and shown on the circle diagram (Figure 3).

The mean value of fold change ratio of each gene between days 1 and 7, 15, and 30 of culture was calculated to identify the most up and down-regulated genes. As a result, we obtained 10 up- and 6 down-regulated genes subjected to further analysis.

**Figure 4:**
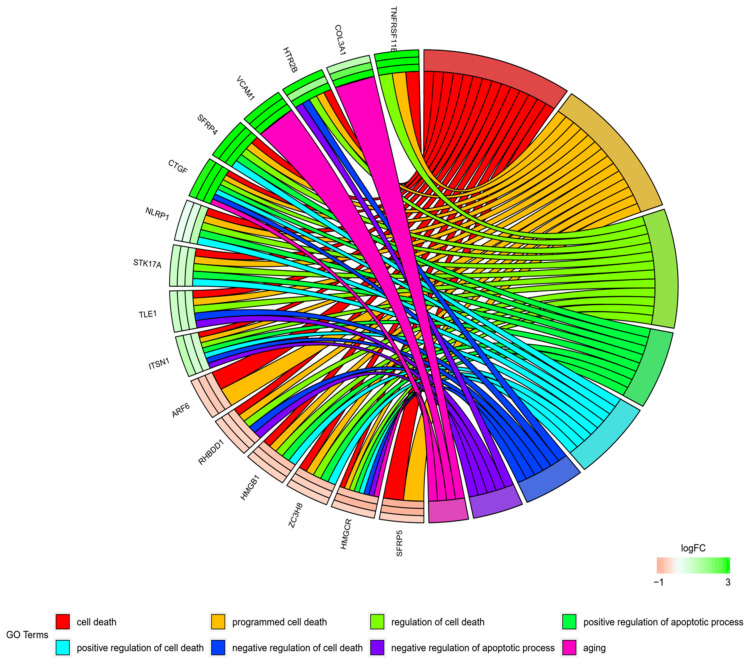
The representation of the mutual relationship of differently expressed genes that belong to 16 chosen genes from “cell death”, “programmed cell death”, “regulation of cell death”, “positive regulation of apoptotic process”, “positive regulation of cell death”, “negative regulation of cell death”, “negative regulation of apoptotic process”, and “aging” GO BP terms. The ribbons indicate which genes belong to which categories. The middle circle represents logarithm from fold change (LogFC) between D7/24 h, D15/24 h, and D30/24 h respectively. The color of each block corresponds to the LogFC of each gene (green—up-regulated, red—down-regulated). The genes were sorted by logFC from most to least changed gene.

**Figure 5:**
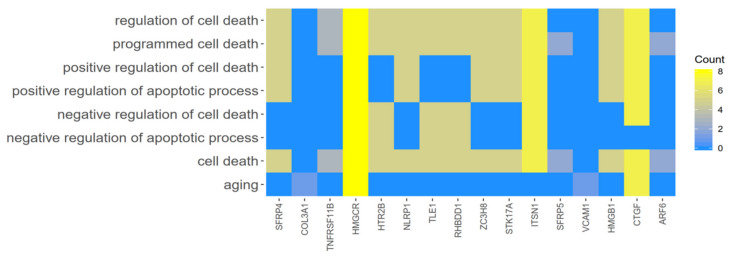
Heatmap showing the gene occurrence between chosen 16 differently expressed genes that belong to “cell death”, “programmed cell death”, “regulation of cell death”, “positive regulation of apoptotic process”, “positive regulation of cell death”, “negative regulation of cell death”, “negative regulation of apoptotic process”, and “aging” GO BP terms. The yellow color is associated with gene occurrence in the GO Term. The intensity of the color corresponds to the number of GO BP terms that each gene belongs to.

**Figure 6:**
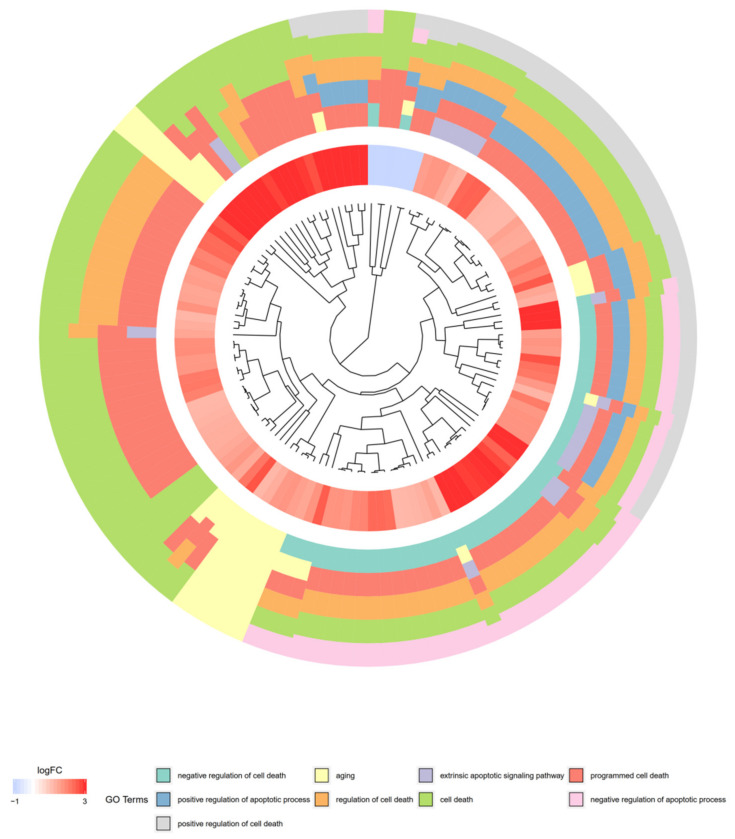
The circular dendrogram of differentially expressed genes involved in “cell death”, “programmed cell death”, “regulation of cell death”, “positive regulation of apoptotic process”, “positive regulation of cell death”, “negative regulation of cell death”, “negative regulation of apoptotic process”, “extrinsic apoptotic signaling pathway”, and “aging” GO BP terms. The genes were clustered based on their logFC values.

Furthermore, to show genes of one GO BP belonging to other categories, their intersections were presented as circle plot (Figure 4), heatmap (Figure 5) as well as a circular dendrogram (Figure 6).

**Figure 7:**
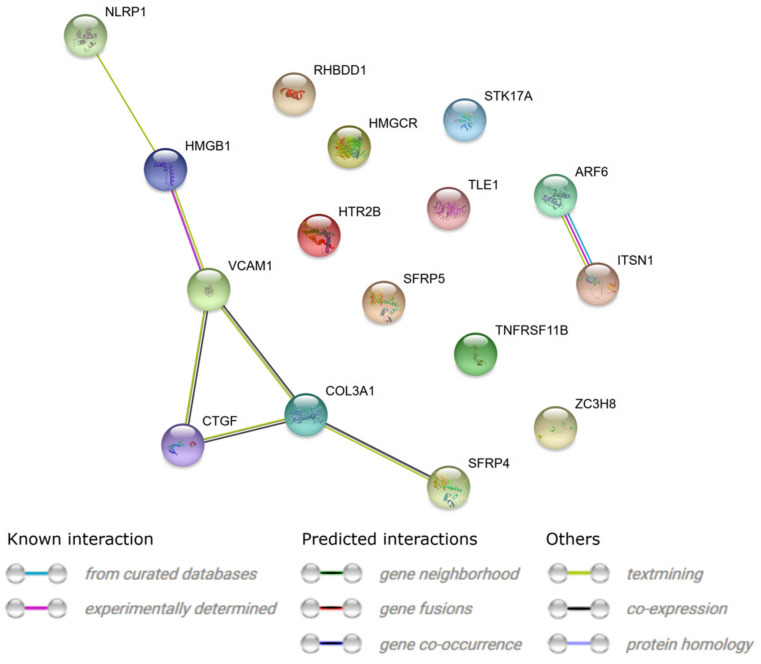
STRING-generated interaction occurrence between 16 chosen differently expressed genes that belongs to the “cell death”, “programmed cell death”, “regulation of cell death”, “positive regulation of apoptotic process”, “positive regulation of cell death”, “negative regulation of cell death”, “negative regulation of apoptotic process”, and “aging” GO BP terms. The intensity of the edges reflects the strength of interaction score.

**Figure 8:**
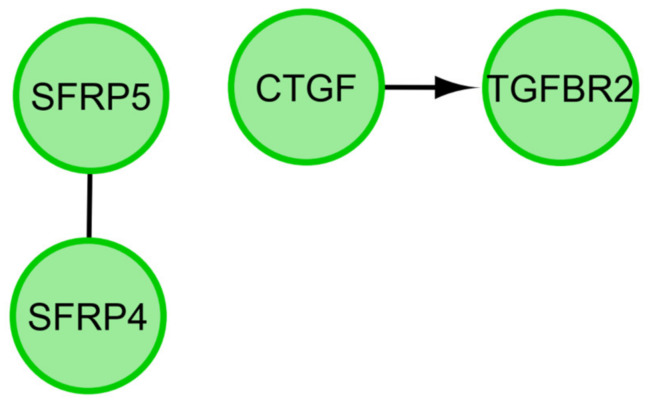
Functional interaction (FI) between 16 chosen differently expressed genes that belongs to the “cell death”, “programmed cell death”, “regulation of cell death”, “positive regulation of apoptotic process”, “positive regulation of cell death”, “negative regulation of cell death”, “negative regulation of apoptotic process”, and “aging” GO BP terms. In the following figure, “–>” stands for activating/catalyzing, “—” for FIs extracted from complexes or inputs.

In the next step, we applied STRING prediction method to generate interaction network among differentially expressed genes of selected GO BPs. Figure 7 presents molecular interaction network formed between protein products of studied genes. Finally, we applied REACTOME FIViz app to Cytoscape 3.6.0 software to investigate the functional interactions between genes of interest and introduced the results in Figure 8.

**Figure 9:**
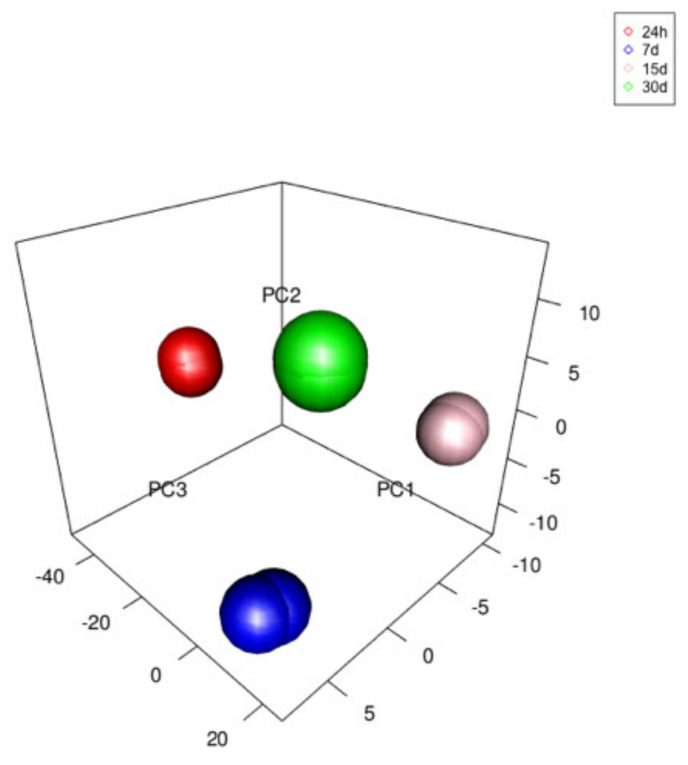
Principal Component Analysis (PCA) plot showing correlations between samples. Samples that are highly correlated cluster together.

In addition, PCA (Principal Component Analysis) analyzes (Figure 9) were performed to illustrate the correlation between samples.

### 3.3. Validation of Gene Expression

**Figure 10:**
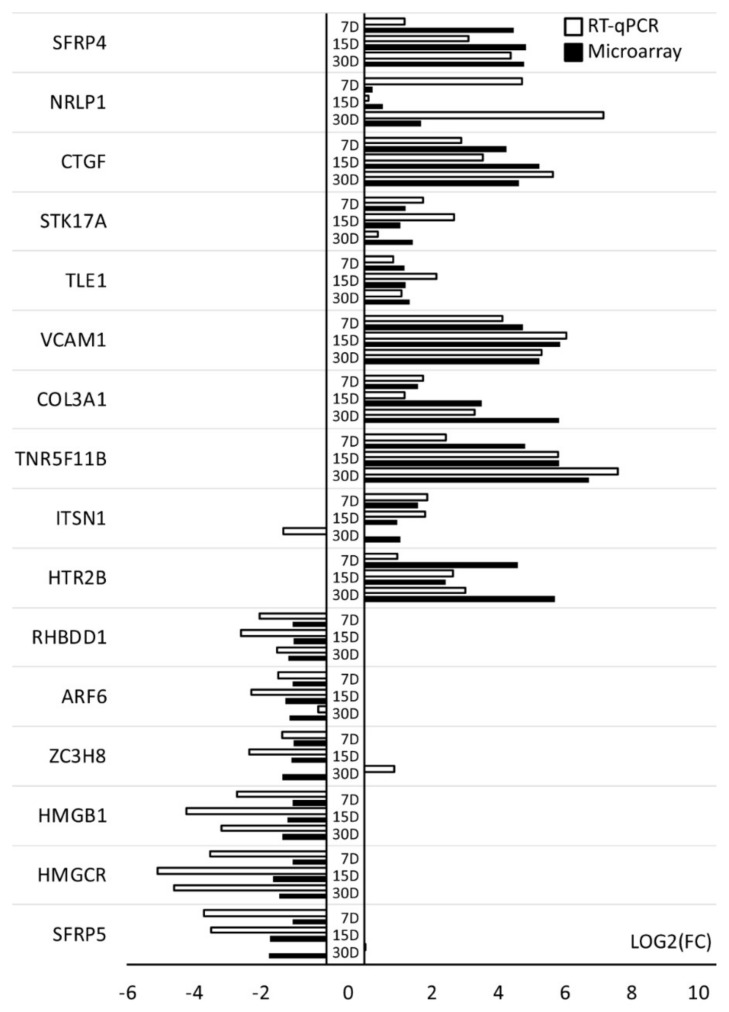
The results of the RT-qPCR validation of expression microarrays (log (FC)), presented as a bar graph. All of the presented sample means were deemed to be statistically significant (*p* < 0.05). D: day of culture; FC: fold change.

The RT-qPCR method was applied in order to quantitatively validate the microarray results. Bar graph (Figure 10) presents validation results.

In the majority of the 16 selected genes, the analysis confirmed the character of expression. As can be seen in most cases, differences do not affect the direction of gene expression. However, as in the case of *ITSN1* and *ZC3H8* genes, some expression pattern disturbances can be observed in one of the analyzed time periods. In such situations, the RT-qPCR result is usually considered more representative, as this method targets the specific sequences with much larger quantitative precision, as opposed to whole transcriptome analysis provided by microarrays. This approach has been also used in the current study, as the difference in sensitivity of the methods can sometimes vary.

## 4. Discussion

During folliculogenesis, the oocyte develops in the presence of cumulus somatic cells (CCs), which support the physiological maturity and capacity to become fertilized [31]. Before folliculogenesis, one of the main CC functions is cyclic guanosine monophosphate (cGMP) production, which is transported through gap junctions (GJs) to the oocyte, inhibiting phosphodiesterase 3A (PDE3A) synthesis. It prevents the hydrolysis of cyclic adenosine monophosphate (cAMP), which is important for maintaining oocyte immaturity at prophase I until recruitment for the process of maturation [32]. CCs are responsible for oocyte contact with the external environment by providing most of the energy substrates. Thus, oocyte competence and ability for further development is mainly dependent on CC regulation [33]. It has been suggested that cumulus cells can maintain their physiological functions and support nuclear and cytoplasmic oocyte maturation for a long time due to their low DNA fragmentation index (DFI) [34]. Decrease in CC apoptosis may result from different survival pathway activation, improving the oocyte quality [35]. Expression of oocyte and CC genes can be altered by changes in their cell cycle and metabolism or induction of apoptosis [36]. Several pro- and anti-apoptotic genes, such as *BCLAF1* and *XAF1,* have been already identified that play an important role in the follicle’s survival, atresia, and apoptosis [37,38].

During gene expression analysis of human ovarian CCs from long-term cultures, particular attention was paid to genes belonging to “Cell death”, “Programmed cell death”, “Regulation of cell death”, “Positive regulation of apoptotic process”, “Positive regulation of cell death”, “Extrinsic apoptotic signaling pathway”, “Negative regulation of cell death”, “Negative regulation of apoptotic process”, and “Aging” ontology groups. Day 30 of cell culture allows us to understand CCs’ properties in in vitro conditions. Day 1 (24 h) analysis corresponds approximately to this cells’ physiological properties, while the following days show the changes that occur in CCs.

The best-known form of PCD in animals is apoptosis. It is characterized by many morphological and biochemical features, which include: cell shrinkage, resulting in mitochondria structure and function damage, cytoplasm contraction, chromatin condensation, and marginalization in the nucleus, as well as DNA and nucleus fragmentation. These processes occur simultaneously, maintaining the integrity of the cell membrane and leading to formation of characteristic bubble-like protrusions described as apoptotic bodies. Cell fragmentation with their formation is the final stage of apoptosis [5,6,13]. In the presented research, CC changed their shape from star-like to slightly elongated, spindle-like during primary in vitro culture. Moreover, it was observed that CCs adhered very quickly to the culture plate bottom. The adhesion of cells to the bottom was observed just a few hours after transfer to the culture plate.

Recent studies show that the expression of genes associated with the process of apoptosis in cumulus cells increases with woman’s age [39,40]. Our study population included patients in the age range comparable to the (younger) group of women in the research presented by Moffatt et al. [39]. These authors noted that a higher expression of apoptotic markers (Fas and TIAR) in CCs of older women may have a detrimental effect on cumulus functionality. These studies also showed a higher level of DNA fragmentation of cumulus cells in the group over 38 years of age. In more recent research, Al-Edani et al. [41] presented an analysis of gene expression in cumulus cells in relation to female age in three categories (below 30, between 31 and 34, and above 37 years of age) demonstrating upregulation of genes associated with angiogenesis in older women. Authors speculated that augmentation of angiogenesis may be a compensatory mechanism to alleviate the deleterious effect of aging and subsequent hypoxia [41].

To assess the level of gene expression, total RNA from primary CCs culture on 1, 7, 15, and 30 days of culture was isolated. From over 133 genes subjected to expression analysis and belonging to the above ontological groups, three gene groups were selected for further study. The first contained *SFRP5*, *HMGCR*, *HMGB1*, *ZC3H8*, *ARF6*, and *RHBDD1* genes whose expression decreased during culture. The second contained *NLRP1*, *ITSN1*, *STK17A,* and *TLE1* genes, classified as having the lowest increase in expression. Finally, the last group included genes with the highest increase in expression during culture, which were *COL3A1*, *SFRP4*, *CTGF*, *HTR2B*, *VCAM1*, *TNFRSF11B* genes. Analysis of biological interactions between genes showed that none of these 16 analyzed genes belonged to the “Extrinsic apoptotic signaling pathway” ontology group.

Among the analyzed genes, the *TNFRSF11B* (tumor necrosis factor receptor superfamily, member 11b) had the highest increase in expression. The protein encoded by this gene belongs to the receptor TNF superfamily and its main role is cell death regulation. This protein can be secreted by osteoblasts, and acts as a negative regulator of bone resorption. Studies in mice have indicated its important role in lymph node organogenesis and vascular calcification [42]. Microarray analysis in mouse ovarian granulosa cell tumors (GCT) revealed the ectopic expression of TNFRSF11B as a bone marker [43].

The second most up-regulated gene is *VCAM1* (vascular cell adhesion molecule 1). In endothelium, activated by cytokines, expression of this gene leads to the production of sialoglycoproteins. Expression of this gene is most often associated with activation of immune responses during inflammation in endothelial cells [44]. VCAM1 is an interstitial progenitor testis cells marker and is present at high levels in adult Leydig cells [45]. In theca cells, increase in VCAM1 expression is preceded by activation of the androgen receptor which indirectly results in change of the expression and/or activity of the *NR2F2* (nuclear receptor subfamily 2, group F, member 2) gene. In mice, this series of reactions prevents androgenization of the ovaries [45]. To date, expression of *TNFRSF11B* and *VCAM1* genes in human cumulus cells has not yet been analyzed.

*HTR2B* also known as 5-hydroxytryptamine (5-HT) receptor 2B encodes one of several types of serotonin receptors. These receptors are thought to be involved in the development of prostate cancer. Serotonin receptors expression has been shown to occur in healthy ovaries as well as in ovarian tumors, indicating its role in both physiological and pathological conditions [46]. To date, there are few reports describing this receptor’s activity in ovarian tissue. Kannisto et al. for the first time presented the importance of 5-HT receptors in bovine ovaries [47]. The first report about 5-HT expression in mammalian oocytes and cumulus cells were presented by Dubé et al. [48]. 5-HT2B receptor, was identified for the first time in mouse cumulus cells [49]. CCs by expressing TPH1 (tryptophan-1 hydroxylase) induce synthesis of HTRB2 ligand—5-HT, which together with other receptors located on the CCs and oocytes surface in the metaphase II (MII) stage triggers dynamic process of Ca^2+^ exchange between these cells. This interaction subsequently leads to effective two-way communication between oocyte and CCs. Given our results and strong *HTR2B* gene expression, this gene seems to be important for Ca^2+^ ion exchange between CCs and the oocyte. It should be emphasized that high concentration of Ca^2+^ ions also plays a major role in oocyte activation at the time of their fertilization [50,51].

*CTGF,* also known as *CCN2* (cellular communication network factor 2), encodes a mitogen secreted by vascular endothelial cells. In ovarian tissue, CCN proteins are regulated by steroid hormones and their expression is dictated by oxygen concentration. CCN2 activity is essential for follicular development, ovulation, and corpus luteum formation, while its inhibition can lead to female infertility. Additionally, acting together with CCN1 and CCN3 by their activity compensation, CCN2 controls human trophoblast proliferation and migration [52]. The increase in *CTGF* gene expression during primary CCs culture observed in our study confirms results of Hillier et al., who similarly noticed increased CCN2 expression during follicular growth and its highest level in COCs [53]. In vivo, CTGF expression is inhibited by follicle stimulating hormone (FSH) and human chorionic gonadotropin (hCG) increase [54]. Studies by Duncan et al. showed that CCN2 is more involved in luteolysis than in luteinization [55]. Given our current and previous results and the results of other researchers, it can be assumed that CCN2 plays a multidirectional role in recruiting primary follicles, their proper development, oocyte maturation, corpus luteum angiogenesis, and luteolysis.

The last two genes analyzed from the group of six with the highest expression are *SFRP4* (secreted frizzled related protein 4) and *COL3A1* (collagen type III alpha 1 chain). SFRP4 participates in modulation of Wnt signaling pathway. In human mural and cumulus granulosa cells, high levels of LH (luteinizing hormone) and hCG in the ovaries reduce their expansion and proliferation as follicles grow to the preovulatory stage. Additionally, it was noted that in CCs SFRP4 expression was higher than in mural GCs [56]. SFRP4 high level is maintained in corpus luteum (CL) confirming previous research describing targeted growth and differentiation of follicular granulosa cells tended to form CL [1]. The role of the *SFRP4* gene in rat CL apoptosis has not been fully determined [57]. Zamberlam et al. for the first time reported that SFRP4 in the presence of gonadotropins negatively regulates the response of ovarian follicles, limiting their development, ovulation, and female fertility [58]. Treatment of human ovaries with recombinant FSH (rFSH) gonadotropin promotes COL3A1 expression in CCs, which is important for cellular assembly and organization [59].

During CCs’ primary cultivation, the lowest level of increased expression was found in *NLRP1* (NLR family pyrin domain containing 1), *ITSN1* (intersectin 1), *STK17A* (serine/threonine kinase 17a), and *TLE1* (TLE family member 1, transcriptional corepressor) genes. The increase in expression of each of these genes was insignificant and on a similar level. *NLRP1* encodes an apoptotic protein belonging to the Ced-4 family and is one of the key programmed cell death mediators. Increased expression of this gene characterizes cells entering the apoptosis pathway. The *NLRP* gene plays a key role in mammalian reproduction. In mice, *NLRP* transcripts are required for successful oocyte development after fertilization [60]. Continuing, ITSN1 participates in mitogenesis and endocytosis regulation. Expression of these two genes in CCs has not yet been studied. The next of four considered genes is *STK17A,* presenting the apoptosis inducing activity. Increased STK17A expression stimulates cell proliferation. Using flow cytometry, an increased percentage of ovarian cancer cells in the G2/M phase was determined while in the G1 phase reduction of cells was observed. Additionally, in ovarian cancer cells, STK17A was found to be characteristic for a drug-resistance phenotype [61]. The last gene from this group—*TLE1* is a specific biomarker of synovial sarcoma and subset of melanomas [62]. The importance and role of this gene in ovarian tissue has not been described. Taking into account minor changes of these genes’ expression and results of previous research, their low importance for cumulus cells can be presumed.

The last of the analyzed groups contains 6 out of all 133 genes whose expression was down-regulated. The most down-regulated gene was *SFRP5* (secreted frizzled related protein 5), whose increased expression was noted in pancreas and retinal pigment epithelium. Unlike *SFRP4*, this gene expression was induced by LH/hCG in CCs and GCs in both in vivo and in vitro conditions [56]. Interestingly, these results indicate that the lack of LH/hCG supplementation in our long-term cultivation reflects the conditions of the initial stages of follicular growth where these hormones are not present. HMGCR (3-hydroxy-3-methylglutaryl-CoA reductase) is a crucial enzyme for cholesterol production. Its significance in ovaries was only considered in the context of ovarian cancer cell line response to cisplatin [63]. The next gene, *HMGB1* (high mobility group box 1) encodes high mobility group-box superfamily protein member. This gene takes part in the cell differentiation and tumor cell migration processes. The role of HMGB1 in promoting apoptosis in some pathological processes has been proven [64]. The research of Xiao Rong et al. showed that this gene may be an ovarian GCs apoptosis extracellular inducer [65]. ARF6 (ADP ribosylation factor 6) is classified as a small GTPase that regulates endocytic membrane distribution and cytoskeleton organization. It localizes at the plasma membrane in the active GTP- and/or inactive GDP-bound form [66]. In GCs, *ARF6* mRNA transcript concentration was shown to be increased in PCOS syndrome. Together with cytohesin2, this gene is involved in LHCGR activity induction [67]. The last two *ZC3H8* (zinc finger CCCH-type containing 8) and *RHBDD1* (rhomboid domain containing 1) genes have not been previously analyzed in the context of ovarian relevance.

## 5. Conclusions

The present study characterized gene expression profiles in cumulus cells during long-term (30 days) in vitro primary culture. All of the studied genes are involved in programmed cell death, especially the apoptosis processes, and belong to the nine ontology groups: cell death (GO:0008219), programmed cell death (GO:0012501), regulation of cell death (GO:0010941), positive regulation of apoptotic process (GO:0043065), positive regulation of cell death (GO:0010942), extrinsic apoptotic signaling pathway (GO:0097191), negative regulation of cell death (GO:0060548), negative regulation of apoptotic process (GO:0043066), and aging (GO:0007568). Detailed analysis of 16 previously selected genes presenting differential expression level allowed us to bring closer the understanding CC aging and cell death. Measurement of gene expression at the first day of culture gives us a picture how expression patterns develop shortly after ovulation. It also enables to trace molecular mechanisms, which are important for CL formation. Among three of the analyzed gene groups, the one including genes with the highest increase in expression seems to be the most crucial for CCs’ functions. This group contains genes directly affecting the CC quality, and through their close communication network, probably indirectly affecting the oocyte. As presented, analyzed genes belong to ontological groups associated with aging and cell death, but it has to be kept in mind that in CCs and ovaries, they may be involved incompletely with distinct physiological functions. Considering the role of Ca^2+^ ions in achieving MII stage by the oocyte and its activation after fertilization, in-depth understanding of the function that this gene can play in CCs may be relevant to understanding of oocyte maturation mechanism. Additionally, Ca^2+^ ions play a key role in the oocyte activation during fertilization. Considering that apoptosis gene expression level changes with the age of the patient, it seems interesting for further research to study expression of CC genes responsible for planned cell death and aging in material obtained from patients of different age ranges. Finally, we have demonstrated that CC long-term in vitro culture is characterized by expression of markers that suggest their ability to differentiate towards corpus luteum cells. Our research can become the basis for creating a model of corpus luteum formation in laboratory conditions.

## Figures and Tables

**Table 1:** Oligonucleotide sequences of primers used for RT-qPCR analysis: the primers of the 10 most up-regulated and 6 most down-regulated genes.

Gene	Primer Sequences (5′-3′)	Product Size (bp)
COL3A1	F: GGGAACAACTTGATGGTGCT R: CCTCCTTCAACAGCTTCCTG	173
SFRP4	F: GCCTGGGACAGCCTATGTAA R: TCTGTACCAAAGGGCAAACC	160
HTR2B	F: GCCTTCTTCACACCTCTTGC R: TGTCCTTTCGAGAACCATCC	199
VCAM1	F: CAGACAGGAAGTCCCTGGAA R: TTCTTGCAGCTTTGTGGATG	212
TNFRSF11B	F: GGCAACACAGCTCACAAGAA R: CTGGGTTTGCATGCCTTTAT	241
TLE1	F: CGACAAGTCCATCAGCAGAA R: CCCAGATCACCCAGAAAGAA	150
STK17A	F: TGAATCTCCATTGGGTGACA R: CCACATATCTGTTGCCATGC	154
ITSN1	F: CTCAGGAAAGGGACAAGCAG R: CTGGCTTAGCTGGTTCTTGG	198
NLRP1	F: ATACTTCCCGAGGCATCCTT R: TTGCTTGCCTTTCATCTGTG	160
RHBDD1	F: AGCTCCTGCCTTAGTGTGGA R: AGCAAATTGCAAGAGCAGGT	225
ARF6	F: GGAAAAGGTGGAGTGGGTTT R: GCACCAACAGGAGCCTACAT	245
ZC3H8	F: CATGCTCCACTGACTCCTGA R: CATCACCCCAGTCACACAAG	215
HMGB1	F: ATATGGCAAAAGCGGACAAG R: GCAACATCACCAATGGACAG	193
HMGCR	F: GTCATTCCAGCCAAGGTTGT R: GGGACCACTTGCTTCCATTA	228
SFRP5	F: TGGAGCCCAGAAAAAGAAGA R: GCAGGGGTAGGAGAACATGA	247
CTGF	F: GGAAAAGATTCCCACCCAAT R: TGCTCCTAAAGCCACACCTT	153
GAPDH	F: TCAGCCGCATCTTCTTTTGC R: ACGACCAAATCCGTTGACTC	90
ACTB	F: AAAGACCTGTACGCCAACAC R: CTCAGGAGGAGCAATGATCTTG	132
HPRT	F: TGGCGTCGTGATTAGTGATG F: ACATCTCGAGCAAGACGTTC	141

**Table 2:** Gene symbols of 16 genes with the largest change expression, Entrez gene IDs, fold change in expression ratio, corrected *p*-values, and mean value of fold change ratio of studied genes.

Gene Symbol	Entrez Gene ID	Ratio 7 d/24 h	Ratio 15 d/24 h	Ratio 30 d/24 h	Adj. *p*-val. 7 d/24 h	Adj. *p*-val. 15 d/24 h	Adj. *p*-val. 30 d/24 h	Mean Ratio
SFRP5	6425	−2.0637	−3.24762	−3.37478	0.000111	3.10∙10^−6^	2.01∙10^−6^	−2.89537
HMGCR	3156	−2.03271	−3.09191	−2.68085	7.50∙10^−6^	2.38∙10^−7^	4.42∙10^−7^	−2.60182
HMGB1	3146	−2.0542	−2.26113	−2.52655	6.96∙10^−6^	2.04∙10^−6^	6.84∙10^−7^	−2.28063
ZC3H8	84524	−2.02274	−2.09189	−2.54816	0.00022	0.000114	2.06∙10^−5^	−2.22093
ARF6	382	−2.04476	−2.3896	−2.1636	1.93∙10^−5^	3.58∙10^−6^	6.57∙10^−6^	−2.19932
RHBDD1	84236	−2.06542	−2.02064	−2.20872	9.65∙10^−5^	8.14∙10^−5^	3.17∙10^−5^	−2.09826
NLRP1	22861	1.187027	1.450696	3.220824	0.164284	0.00633	4.21∙10^−6^	1.952849
ITSN1	6453	3.052942	1.989949	2.094111	6.63∙10^−5^	0.000968	0.000558	2.379001
STK17A	9263	2.316524	2.118637	2.740376	8.16∙10^−6^	1.15∙10^−5^	1.29∙10^−6^	2.391846
TLE1	7088	2.291749	2.358883	2.555574	3.64∙10^−6^	1.98∙10^−6^	8.50∙10^−7^	2.402069
COL3A1	1281	2.994386	11.33677	56.74367	1.93∙10^−6^	9.27∙10^−9^	7.86∙10^−10^	23.69161
SFRP4	6424	22.1843	28.77543	27.23046	5.34∙10^−8^	1.93∙10^−8^	1.44∙10^−8^	26.06339
CTGF	1490	18.97488	37.72449	24.63015	3.75∙10^−9^	6.96∙10^−10^	7.86∙10^−10^	27.10984
HTR2B	3357	24.3271	5.361072	52.86955	4.41∙10^−8^	1.50∙10^−6^	4.69∙10^−9^	27.51924
VCAM1	7412	26.68641	58.39253	38.13633	3.75∙10^−9^	6.96∙10^−10^	7.86∙10^−10^	41.07175
TNFRSF11B	4982	28.13419	57.62667	105.3113	3.53∙10^−8^	6.38∙10^−9^	2.12∙10^−9^	63.69073

## Supplementary Materials

The following are available online at https://www.mdpi.com/2073-4409/9/5/1265/s1, Table S1: List of the selected 133 genes.
