# Supplementary material for: Human Cumulus Cells in Long-Term In Vitro Culture Reflect Differential Expression Profile of Genes Responsible for Planned Cell Death and Aging—A Study of New Molecular Markers
Source: Cells. 2020 May 21;9(5):1265. doi: 10.3390/cells9051265 (PMC7291080; doi:10.3390/cells9051265)
Supplement: Supplementary file 1 [file cells-09-01265-s001.pdf]

Supplementary Table 1. List of the selected 133 genes.

Gene symbol.	Entrez gene ID	Ratio 7d/24 h	Ratio 15d/24 h	Ratio 30d/24 h	Adj. p-val. 7d/24 h	Adj. p-val. 15d/24 h	Adj. p-val. 30d/24 h	Mean ratio
SFRP5	6425	-2,06370	-3,24762	-3,37478	0,000111	3,10E-06	2,01E-06	-2,89537
HMGCR	3156	-2,03271	-3,09191	-2,68085	7,50E-06	2,38E- 07	4,42E-07	-2,60182
HMGB1	3146	-2,05420	-2,26113	-2,52655	6,96E-06	2,04E-06	6,84E-07	-2,28063
ZC3H8	84524	-2,02274	-2,09189	-2,54816	0,00022	0,000114	2,06E-05	-2,22093
ARF6	382	-2,04476	-2,38960	-2,16360	1,93E-05	3,58E-06	6,57E-06	-2,19932
RHBDD1	84236	-2,06542	-2,02064	-2,20872	9,65E-05	8,14E-05	3,17E-05	-2,09826
NLRP1	22861	1,18703	1,45070	3,22082	0,164284	0,00633	4,21E-06	1,952849
ITSN1	6453	3,05294	1,98995	2,09411	6,63E-05	0,000968	0,000558	2,379001
STK17A	9263	2,31652	2,11864	2,74038	8,16E-06	1,15E-05	1,29E-06	2,391846
TLE1	7088	2,29175	2,35888	2,55557	3,64E-06	1,98E-06	8,50E-07	2,402069
GDF11	10220	2,99003	2,36048	2,00660	2,56E-06	8,71E-06	2,98E-05	2,452372
MET	4233	2,57716	2,55336	2,28941	4,71E-06	3,39E-06	6,28E-06	2,47331
UACA	55075	2,28171	2,03435	3,30500	4,07E-06	7,32E-06	1,78E-07	2,540354
TIMP2	7077	2,57040	2,18627	3,02322	1,71E-06	4,02E-06	3,13E-07	2,593296
DMD	1756	2,29376	3,10211	2,44562	8,68E-06	7,75E-07	2,90E-06	2,613829
PLAC8	51316	3,42485	2,33403	2,09272	1,43E-05	0,000112	0,000235	2,617201
RAB7L1	8934	2,65412	2,55260	2,65651	9,43E-07	8,49E-07	4,85E-07	2,621075
SP100	6672	2,77029	2,21668	2,92318	7,52E-07	2,59E-06	2,77E-07	2,636717
WFS1	7466	2,79389	2,78393	2,34564	4,91E-07	3,40E-07	8,81E-07	2,641152
DNASE2	1777	2,39881	2,57242	2,99500	5,44E-06	2,19E-06	6,38E-07	2,655413
IL12A	3592	3,27581	2,54814	2,32292	3,15E-05	0,000101	0,000171	2,715623
EDA2R	60401	2,33245	3,52833	2,40926	1,85E-06	9,74E-08	7,68E-07	2,756678
OPTN	10133	2,66917	2,59473	3,09579	7,25E-06	5,90E-06	1,52E-06	2,786564
CREB3L1	90993	2,45605	2,47671	3,52275	2,97E-06	1,91E-06	1,63E-07	2,818506
RTN4	57142	2,33093	3,39554	2,78007	2,39E-06	1,51E-07	3,63E-07	2,835513
PEG10	23089	2,54295	3,78459	2,38662	2,90E-06	1,94E-07	2,53E-06	2,904719
TLR2	7097	2,57632	3,21800	2,94372	2,44E-05	4,08E-06	5,69E-06	2,91268
DUSP6	1848	2,89364	2,64440	3,45628	3,10E-05	3,75E-05	6,18E-06	2,998105
XAF1	54739	4,07885	2,18562	2,75805	2,49E-07	7,71E-06	1,08E-06	3,007508
PEA15	8682	2,35707	2,99411	3,78280	2,28E-06	3,09E-07	6,57E-08	3,044662
RAC1	5879	2,62403	3,68475	2,83597	0,00024	2,36E-05	8,78E-05	3,048248
GADD45B	4616	2,29549	3,23499	3,62059	7,37E-06	5,16E-07	2,10E-07	3,050357
DAPK1	1612	3,42703	3,17152	2,69085	2,12E-07	2,11E-07	4,42E-07	3,096469
SERPINE1	5054	2,29595	2,98828	4,04130	8,61E-06	9,69E-07	1,40E-07	3,108508
KIF14	9928	3,88656	3,45911	2,03914	3,06E-07	3,67E-07	1,20E-05	3,128268
EPHA2	1969	3,26551	4,03620	2,22666	2,68E-06	6,36E-07	2,06E-05	3,176124
TRAF5	7188	3,10255	3,77083	3,11043	3,72E-05	8,79E-06	2,11E-05	3,327935
IFI27	3429	3,25013	2,87110	3,99654	6,07E-07	8,82E-07	1,06E-07	3,372587
RGS20	8601	3,25370	2,23282	4,64785	4,43E-06	3,77E-05	4,01E-07	3,378124
EEF1A2	1917	2,39363	4,86449	3,01890	0,001405	2,30E-05	0,000205	3,42567
PLK2	10769	2,35362	3,14767	4,87360	2,10E-06	2,12E-07	1,94E-08	3,458295
TGFA	7039	2,25652	3,10430	5,05508	0,000523	4,47E-05	3,42E-06	3,471968
DAPK3	1613	2,63253	4,96231	2,94553	5,30E-06	1,34E-07	1,35E-06	3,513455
SORT1	6272	3,03901	3,79547	3,73018	5,25E-07	1,14E-07	8,59E-08	3,521552
ETS1	2113	2,82960	3,53666	4,33193	7,77E-07	1,51E-07	4,22E-08	3,566063
IRF1	3659	3,65168	4,04743	3,05897	2,40E-06	1,02E-06	3,54E-06	3,586026
CD14	929	2,51385	5,43572	2,81739	1,19E-05	1,51E-07	3,04E-06	3,58899
APBB2	323	3,19251	4,56494	3,01512	9,62E-07	1,16E-07	6,97E-07	3,590857
TSPO	706	2,77299	4,00989	4,07616	1,80E-06	1,65E-07	1,10E-07	3,619678
TRIO	7204	3,89782	3,39290	3,84140	1,33E-06	1,84E-06	7,56E-07	3,710704
NEK6	10783	2,65055	3,99543	4,60990	2,28E-06	1,59E-07	6,18E-08	3,751958

CYR61	3491	3,36179	4,30449	3,66318	4,72E-07	9,89E-08	1,49E-07	3,776483
FAM134B	54463	2,59545	4,05822	4,77310	3,17E-06	1,74E-07	6,30E-08	3,808924
IDO1	3620	6,30239	2,78159	2,43391	1,39E-07	3,93E-06	8,22E-06	3,839298
PYCARD	29108	2,38530	4,69852	4,46127	0,001323	2,43E-05	2,59E-05	3,848364
SQSTM1	8878	4,48457	2,70719	4,56235	9,80E-08	8,49E-07	3,95E-08	3,918037
NGF	4803	4,34691	2,75855	4,82541	1,91E-06	1,50E-05	6,48E-07	3,976955
HMGA1	3159	3,18606	2,73842	6,06702	4,04E-07	7,03E-07	1,19E-08	3,997166
BMP1	649	2,77541	4,44392	5,01760	4,87E-07	3,07E-08	1,28E-08	4,078977
SOX4	6659	3,26313	5,73266	4,02026	2,04E-07	1,28E-08	3,43E-08	4,338685
SFRP1	6422	3,40317	5,84430	3,87700	1,72E-05	1,06E-06	4,89E-06	4,374824
GAS1	2619	3,98269	5,15983	4,36167	2,38E-06	5,39E-07	8,39E-07	4,501396
DPYSL4	10570	2,91729	7,24730	3,37933	1,14E-06	1,61E-08	2,52E-07	4,514641
CAMK2D	817	5,55329	4,19353	3,89132	1,93E-07	3,96E-07	4,32E-07	4,546045
TGFBR1	7046	3,26648	3,38315	7,27426	9,18E-07	5,16E-07	1,62E-08	4,641299
BOK	666	3,27736	6,49404	4,31952	1,15E-06	4,22E-08	1,45E-07	4,696971
TLR4	7099	4,45863	4,47094	5,38563	1,32E-07	8,27E-08	2,74E-08	4,77173
IFI6	2537	5,50438	2,86910	5,99548	1,17E-07	1,63E-06	3,84E-08	4,789654
CAV1	857	3,40282	5,86552	5,26002	2,02E-06	1,30E-07	1,39E-07	4,842787
ZNF385D	79750	4,40250	4,64457	5,49415	2,05E-07	1,10E-07	4,02E-08	4,84707
ICAM1	3383	3,21699	3,54526	7,78238	3,38E-07	1,39E-07	5,02E-09	4,848208
TWIST1	7291	5,80259	6,00426	2,78705	6,97E-08	3,90E-08	1,07E-06	4,864636
STAT1	6772	5,92495	5,33765	3,38607	5,83E-08	4,99E-08	2,83E-07	4,882889
IGFBP3	3486	2,28321	6,72824	6,21667	0,000651	2,01E-06	2,13E-06	5,076038
IFIT3	3437	7,84232	3,52693	3,95586	1,12E-08	1,03E-07	4,22E-08	5,108367
F3	2152	4,39767	6,41967	4,67450	1,15E-07	1,76E-08	3,93E-08	5,163944
DEPTOR	64798	5,12412	5,40605	4,97199	5,77E-08	2,78E-08	2,65E-08	5,167387
NDRG1	10397	3,41184	7,61826	4,71913	3,00E-06	7,97E-08	3,35E-07	5,249743
EDN1	1906	2,75473	6,25150	6,75370	9,24E-06	1,32E-07	7,15E-08	5,253311
PRKAA2	5563	3,25709	7,67613	4,99005	1,94E-05	3,61E-07	1,36E-06	5,307757
IER3	8870	3,99456	4,91432	7,04098	1,59E-07	4,18E-08	8,22E-09	5,316621
TNFRSF21	27242	5,19937	5,20060	6,56186	3,48E-07	2,37E-07	7,29E-08	5,653944
KDR	3791	3,37633	3,48932	10,93479	2,87E-05	1,62E-05	1,67E-07	5,933482
DFNA5	1687	4,16396	5,86332	7,84651	1,10E-07	1,82E-08	5,07E-09	5,95793
SERPINB9	5272	7,29692	6,40547	4,70991	1,54E-07	1,49E-07	3,64E-07	6,137435
FMN2	56776	5,20459	4,90735	8,34569	1,80E-06	1,57E-06	1,76E-07	6,152541
FGF2	2247	4,55154	4,29011	10,23021	7,19E-08	5,70E-08	2,63E-09	6,357287
LOXL2	4017	3,35396	8,68753	7,97981	2,09E-07	4,70E-09	3,80E-09	6,673768
STEAP3	55240	6,79768	6,12528	7,12268	6,46E-08	5,44E-08	2,28E-08	6,681879
CDKN2A	1029	2,48203	5,31878	12,66319	2,16E-06	3,07E-08	2,04E-09	6,821333
APLP1	333	3,24659	8,55481	8,73928	4,40E-06	6,11E-08	4,07E-08	6,846891
ACTN1	87	4,28986	9,80829	6,95764	7,91E-07	3,11E-08	6,18E-08	7,018597
CTSC	1075	5,92580	8,48329	7,74530	2,39E-08	4,72E-09	3,91E-09	7,384799
LOC728392	22861	4,63695	9,59466	8,62165	4,55E-07	2,70E-08	2,56E-08	7,617752
SRGN	5552	3,59427	3,36555	16,03813	4,61E-06	4,39E-06	1,49E-08	7,665985
ARNT2	9915	7,57284	7,12031	8,37831	2,15E-08	1,34E-08	5,52E-09	7,690486
LITAF	9516	6,22046	5,88639	11,90545	4,96E-08	3,40E-08	3,42E-09	8,004099
LYN	4067	5,30837	5,89434	13,48571	1,08E-07	4,51E-08	3,42E-09	8,229475
TGM2	7052	4,76505	4,05343	16,25102	4,09E-08	4,36E-08	8,41E-10	8,3565
AKR1C3	8644	5,47712	4,48185	15,56029	7,67E-08	1,05E-07	2,12E-09	8,506421
GHR	2690	7,17051	12,18457	6,33522	2,13E-08	3,43E-09	1,04E-08	8,563433
MX1	4599	19,60296	2,99655	3,54938	3,21E-09	2,11E-07	6,18E-08	8,716299
GREM1	26585	5,03946	7,53198	13,94377	3,20E-08	5,09E-09	9,58E-10	8,838402
CD44	960	5,23506	7,51377	14,00705	1,80E-07	3,42E-08	4,71E-09	8,918627
F2R	2149	8,21782	9,37087	10,18710	8,39E-07	3,80E-07	2,26E-07	9,258596
EMP1	2012	8,09990	5,26101	14,48589	3,23E-08	7,25E-08	3,02E-09	9,282268
CCL2	6347	3,38654	13,25710	11,61219	1,04E-06	6,72E-09	6,00E-09	9,418609

DAB2	1601	6,91594	9,34702	13,31417	2,45E-08	6,01E-09	1,98E-09	9,859042
BMP4	652	10,74460	10,66137	9,30856	2,45E-08	1,41E-08	1,33E-08	10,23818
ANXA1	301	9,59325	8,06364	14,17340	1,84E-08	1,49E-08	2,69E-09	10,6101
PHLDA2	7262	6,45156	11,07072	14,51824	9,85E-06	1,26E-06	4,79E-07	10,68017
GULP1	51454	10,77555	7,70504	15,18104	2,39E-08	3,07E-08	4,04E-09	11,22054
ANKRD1	27063	19,28508	9,50711	5,02056	5,39E-09	8,79E-09	4,63E-08	11,27092
IFIT2	3433	10,72331	13,21470	11,96318	7,72E-09	3,04E-09	2,04E-09	11,96706
KRT18	3875	14,55411	15,33562	7,49976	8,40E-09	4,13E-09	1,39E-08	12,46316
CASP1	834	12,24412	9,40038	21,97880	1,48E-08	1,36E-08	1,60E-09	14,5411
KRT8	3856	16,03768	19,91459	8,69636	1,39E-07	5,46E-08	3,26E-07	14,88288
USP53	54532	12,00398	15,08157	18,25725	6,21E-07	2,38E-07	1,11E-07	15,11426
GAS6	2621	8,02323	18,05603	21,07508	4,64E-08	4,45E-09	2,12E-09	15,71812
NTF3	4908	10,24741	17,69989	20,61960	3,93E-07	7,09E-08	3,64E-08	16,18896
CARD16	114769	10,68154	17,11509	24,98621	7,87E-08	1,61E-08	5,20E-09	17,59428
PRUNE2	158471	7,62169	19,74470	31,39542	6,69E-08	4,70E-09	1,40E-09	19,58727
DLC1	10395	2,64410	7,93585	50,13780	3,20E-06	1,76E-08	7,86E-10	20,23925
IGFBP1	3484	23,10358	21,53218	16,27355	3,32E-08	2,11E-08	2,63E-08	20,3031
SEMA5A	9037	13,15295	27,00098	27,70650	7,51E-09	1,70E-09	8,41E-10	22,62015
TGFBR2	7048	15,71098	14,21048	38,01073	5,39E-09	3,04E-09	7,13E-10	22,64407
CD74	972	14,17717	28,19624	27,18397	1,16E-08	2,65E-09	1,28E-09	23,18579
COL3A1	1281	2,99439	11,33677	56,74367	1,93E-06	9,27E-09	7,86E-10	23,69161
SFRP4	6424	22,18430	28,77543	27,23046	5,34E-08	1,93E-08	1,44E-08	26,06339
CTGF	1490	18,97488	37,72449	24,63015	3,75E-09	6,96E-10	7,86E-10	27,10984
HTR2B	3357	24,32710	5,36107	52,86955	4,41E-08	1,50E-06	4,69E-09	27,51924
VCAM1	7412	26,68641	58,39253	38,13633	3,75E-09	6,96E-10	7,86E-10	41,07175
TNFRSF11B	4982	28,13419	57,62667	105,31132	3,53E-08	6,38E-09	2,12E-09	63,69073
